# HARMONIZATION OF SELF-REPORTED RACE/ETHNICITY TO ENHANCE HEALTH EQUITY RESEARCH USING MEDICARE DATA

**DOI:** 10.1093/geroni/igad104.1464

**Published:** 2023-12-21

**Authors:** Anum Zafar, Olga Jarrín, Hyosin Kim, Soko Setoguchi, Marie Harvey, Bei Wu, Maria Lopez, Haiqun Lin

**Affiliations:** Rutgers, The State University of New Jersey, New Brunswick, New Jersey, United States; Rutgers, The State University of New Jersey, New Brunswick, New Jersey, United States; Oregon State University, Corvallis, Oregon, United States; Rutgers, The State University of New Jersey, New Brunswick, New Jersey, United States; Oregon State University, Corvallis, Oregon, United States; New York University, New York City, New York, United States; Rutgers, The State University of New Jersey, New Brunswick, New Jersey, United States; Rutgers, The State University of New Jersey, Newark, New Jersey, United States

## Abstract

Collection of valid race and ethnicity data is critical to understanding and promoting health equity. However, current administrative data often misclassifies members of the racial/ethnic minority groups. To address this issue, the Research Triangle Institute (RTI) developed an imputed race variable, which improves on administrative race data for Hispanic and Asian beneficiaries, but still has limitations. This study compares the RTI race with our new, harmonized, self-reported race/ethnicity data collected during Medicaid enrollment and post-acute and long-term care assessments. Out of 2,322,071 Medicare beneficiaries aged 18 and older who died in 2019 in the U.S., self-reported race/ethnicity was available for 75.1% (1,745,327) who formed the study population. Compared to the RTI race variable, our new race variable categorized more Medicare beneficiaries as non-Hispanic white (79.5% vs. 77.9%) and Asian American/Pacific Islander (2.5% vs. 2.3%), a similar percentage as American Indians/Alaska Native (0.5% vs. 0.5%), and slightly fewer as Black (11.1% vs. 10.9%) and Hispanic (7.2% vs. 6.5%). Using our new race variable as the gold-standard, RTI race had good validity (k=0.81), however sensitivity was highest for non-Hispanic white (96.5) and Black (95.8) beneficiaries, and lower for Hispanic (87.2), Asian American/Pacific Islander (76.2), and American Indian/Alaska Native (58.7) beneficiaries. These findings are consistent with prior work, comparing RTI race with self-reported race from a single data source. Our study provides empirical evidence to support the U.S. Office of the Inspector General’s recommendations to the Centers for Medicare and Medicaid Services to incorporate self-reported race/ethnicity data available to reduce error and bias.

